# Supplementary material for: Adoption of AI writing tools among academic researchers: A Theory of Reasoned Action approach
Source: PLoS One. 2025 Jan 9;20(1):e0313837. doi: 10.1371/journal.pone.0313837 (PMC11717249; doi:10.1371/journal.pone.0313837)
Supplement: S1 Appendix — (DOCX) [file pone.0313837.s002.docx]

**Appendix:**

Questionnaire:

| **Career Stage** | Early Career (Grad Student/Post-doc/Junior Faculty) | | | | | | [67] |
| --- | --- | --- | --- | --- | --- | --- | --- |
|  | Mid Career (Advanced Assistant/Associate Professor) | | | | | |  |
|  | Late Career (Senior Faculty/Professor) | | | | | |  |
|  | Emeritus Professor | | | | | |  |
| **Academic Discipline** | Physical Sciences (Physics, Chemistry, etc.) | | | | | | By Authors |
|  | Social Sciences (Psychology, Sociology, etc.) | | | | | |  |
|  | Arts & Humanities | | | | | |  |
|  | Health Sciences | | | | | |  |
|  | Business/Management Fields | | | | | |  |
|  | Engineering/Computer Science | | | | | |  |
| **Familiarity with AI Tools** | Before taking this survey, my familiarity level with artificial intelligence writing technologies was: | No Familiarity | Low Familiarity | Medium Familiarity | High Familiarity | Very High Familiarity | [35] |
| **Prior Usage /Experience** | The past year, how often have you used some form of AI technology to assist with your academic writing? | Never | Once or Twice | Monthly | Weekly | Daily | By Authors |
|  | I have prior experience using the following AI writing tools or assistants (Select all that apply) | None | Writing Suggestions in Word Processing Programs | Automated Grammar Checking | Research Paper Editing Tools | ChatGPT | Research Article Summarization Tools |
| **Variable** | **Items** | **1** | **2** | **3** | **4** | **5** | **Prior Studies** |
| **Intentions to Use AI Writing Tools** | 1. I intend to use an AI writing assistant the next time I work on a research paper, manuscript, or other academic document. | Strongly Disagree | Disagree | Neutral | Agree | Strongly Agree | [27,35] |
|  | 2. I plan to utilize AI writing tools to help me with certain elements of my academic writing over the next year. | Very Unlikely | Unlikely | Neither Likely nor Unlikely | Likely | Very Likely |  |
|  | 3. If I had access to a high-quality AI academic writing assistant, I would use it to co-write some components of my next research paper. | Strongly Disagree | Disagree | Neutral | Agree | Strongly Agree |  |
|  | 4. My goal is to incorporate AI writing assistants into multiple stages of developing research manuscripts and reports within the next 2 years. | Not at All My Goal | Low Priority Goal | Medium Priority Goal | High Priority Goal | My Top Priority Goal |  |
|  | 5. I want to try using AI text generators and writing assistants to support improving my academic writing productivity and quality. | Does Not Describe Me | Describes Me Slightly | Describes Me Somewhat | Describes Me Well | Describes Me Perfectly |  |
|  | 6. Within your expected upcoming papers, what proportion do you intend to write using assistance from an AI writing tool? | 0% | 25% | 50% | 75% | 100% |  |
| **Attitudes Towards AI Writing Tools** | 1. Using an AI writing assistant to help craft elements of my academic writing would be: | Very Bad Practice | Bad Practice | Neutral Practice | Good Practice | Very Good Practice | [67] |
|  | 2. Writing papers with support from an AI tool seems like it would be a ____ experience for me: | Very Negative | Negative | Neutral | Positive | Very Positive |  |
|  | 3. Using AI technologies to augment my academic writing would likely be: | Very Harmful | Harmful | Neither Harmful nor Beneficial | Beneficial | Very Beneficial |  |
|  | 4. I would describe my overall attitudes towards utilizing AI assisted writing as: | Very Unfavorable | Unfavorable | Neutral | Favorable | Very Favorable |  |
|  | 5. When I think about relying on AI writing assistants, I feel ____ about incorporating them into my workflow: | Very Apprehensive | Apprehensive | Neither Apprehensive nor At Ease | At Ease | Very At Ease |  |
| **Perceived Subjective Norms** | 1. Many of my academic peers think using AI tools to enhance writing is a favorable practice. | Strongly Disagree | Disagree | Neutral | Agree | Strongly Agree | [35] |
|  | 2. Researchers I know have been positive when discussing adopting AI writing assistants. | Definitely False | FALSE | Neither False nor True | TRUE | Definitely True |  |
|  | 3. My colleagues encourage usage of new technologies like AI writing tools to boost research productivity. | Not at All | A Little | Somewhat | A Lot | Very Much So |  |
|  | 4. In my academic circles and institutions, using AI writing assistants is viewed as: | Very Inappropriate | Inappropriate | Neither Inappropriate nor Appropriate | Appropriate | Very Appropriate |  |
|  | 5. The researchers I collaborate with would ____ of me using an AI assistant for help with my academic writing when needed. | Strongly Disapprove | Disapprove | Neither Disapprove nor Approve | Approve | Strongly Approve |  |
| **Perceived Barriers** | 1. I am concerned about the potential high costs of accessing quality AI writing tools and assistants. | Strongly Disagree | Disagree | Neutral | Agree | Strongly Agree | Wang & Cheng (2021), |
|  | 2. Not having access to the computational resources and infrastructure to run AI writing tools would prevent me from adopting their use. | Definitely False | FALSE | Neither False nor True | TRUE | Definitely True |  |
|  | 3. I am uncertain if my institution would fund access to AI assisted writing technologies. | Very Unlikely | Unlikely | Neither Unlikely nor Likely | Likely | Very Likely |  |
|  | 4. Lacking the technical skills to use AI writing tools would be a major barrier for me to incorporate them into my workflow. | Not at all a Barrier | A Minor Barrier | A Moderate Barrier | A Major Barrier | A Very Much a Barrier |  |
|  | 5. Needing to get my institution’s IT department to approve and support new software hinders my adoption of emerging writing technologies like AI. | Strongly Disagree | Disagree | Neutral | Agree | Strongly Agree |  |
|  | 6. I am concerned that legal or ethical risks around AI writing tools could limit my willingness to utilize them. | Does not Describe Me | Describes Me Slightly | Describes Me Somewhat | Describes Me Well | Describes Me Perfectly |  |
|  | 7. Not knowing enough about how to properly cite or acknowledge AI support poses a challenge to me adopting AI writing assistants. | Very Unimportant | Unimportant | Neither Unimportant nor Important | Important | Very Important |  |
