# Supplementary material for: Adoption of AI writing tools among academic researchers: A Theory of Reasoned Action approach
Source: PLoS One. 2025 Jan 9;20(1):e0313837. doi: 10.1371/journal.pone.0313837 (PMC11717249; doi:10.1371/journal.pone.0313837)
Supplement: Attachment — Submitted filename: Responses to the Manuscript from the academic edit.docx [file pone.0313837.s003.docx]

Responses to the Manuscript from the academic editor

Abstract

The study clearly demonstrates the impact of attitudes and subjective norms on AI writing tool adoption but could benefit from a deeper exploration of the perceived barriers. Also, the use of Structural Equation Modeling (SEM) is appropriate; however, additional details on sample selection and its impact on generalizability would strengthen the methodology. Finally, practical implications are well presented, but providing concrete examples of strategies to promote adoption would enhance the study's relevance to stakeholders.

Introduction

The introduction effectively sets the context for studying AI writing tool adoption among academic researchers, but it could benefit from clarifying the unique contributions of TRA compared to UTAUT in this research. While the background on AI writing tools is comprehensive, providing more recent statistics on their usage in academia would strengthen the relevance of the study. However, the study's contributions are well outlined, but the introduction would be improved by offering a clearer statement of the specific research questions or hypotheses guiding the study and should be clearly statement towards the end of the introductory section.

Literature Review

The discussion provides a thorough overview of TRA and related technology adoption models but could benefit from a clearer justification for why TRA was chosen over TAM or UTAUT for this specific study. Also, while the section effectively contextualizes TRA within existing research, it would be strengthened by offering more examples of how TRA has been specifically applied to AI tools in academic or professional contexts. The comparison of different technology acceptance models is comprehensive; however, consolidating some references could improve readability and flow. Finally, it would be better to merge Theory of Reasoned Action and related technologies to improve flow while reading.

Methodology

The use of a purposive sampling strategy is appropriate, but the methodology section could benefit from a more detailed rationale for why this sampling method was chosen over other approaches.

The sample size determination and power analysis are well-explained; however, a brief discussion on how the final sample of 150 compares with similar studies in terms of generalizability would be helpful. The survey instrument is well-developed, but the section could be strengthened by providing specific examples of how feedback from the pre-test was incorporated into the final questionnaire.

The use of various established scales for measuring the TRA constructs is commendable. However, including a brief discussion on the process of adapting these scales to the context of AI writing tools would enhance the understanding of the instrument’s relevance. The methodology section could benefit from a clearer explanation of the procedures for ensuring data quality and handling incomplete responses, beyond just the reminder emails and response rate

Results

However, H2 proposing that perceived barriers negatively shape attitudes was not statistically supported. The estimated negative effect of barriers on tool perceptions (β=0.085; p=0.298) was inadequate to infer significance. Consequently, the subsequent mediation chain suggested in H3 of barriers dampening intentions via attitudes, also found no empirical validation (β=-0.107; p=0.136).

Technically, it would be helpful to clarify that the non-significant results indicate a lack of evidence to support these hypotheses, and hence, these pathways do not contribute to the model as initially proposed. Rewriting the section in a simple way (readable) might improve clarity

Also, this --- Lastly, H6 forwarded higher barriers weakening peer support across academia environment.Analysis corroborated this presumption as well with barriers linked to reducing normative approval (β=0.440; p<0.001). Rewriting the section in a simple way (readable) might improve clarity

Also, the SRMR values for both the saturated and estimated models are reported as 0.093, indicating a moderate fit. While these values are slightly above the ideal threshold of 0.08, they are consistent across different confidence levels. This suggests that the estimated model does not offer a significant improvement in fit compared to the saturated model. It would be beneficial to consider additional fit indices or refine the model to achieve a better fit.

Discussion

The discussion effectively highlights the strong influence of attitudes and subjective norms on adoption intentions. However, a more detailed exploration of why perceived barriers did not significantly impact attitudes or intentions could enhance the analysis.

The section provides valuable insights into the model’s explanatory power and predictive validity. To further strengthen the discussion, consider comparing these findings with similar studies in the AI adoption domain to contextualize the TRA model's performance. The implications for practice are well-articulated, but adding specific examples of how tool developers and academic institutions can implement strategies based on the findings would provide more actionable guidance. Please, separating the discussion from the implications can improve clarity and focus.

Conclusion

The conclusion effectively summarizes the main findings of the study but could benefit from a clearer presentation of the key results. Consider explicitly stating the most significant findings at the beginning of the conclusion to ensure readers can easily grasp the core contributions of the study.

The discussion of practical implications is insightful, but it could be strengthened by providing more specific recommendations for stakeholders. For example, elaborating on actionable strategies for tool developers, academic institutions, and publishers based on the study’s findings would offer more concrete guidance for promoting AI writing tool adoption.

While the limitations are acknowledged, the conclusion would benefit from a more detailed discussion of potential future research directions. Providing specific examples of additional variables or methodologies that could address the study’s limitations would enhance the practical utility of the conclusion for guiding future investigations in this area

**Improve on the manuscript’s grammar and syntax**

The conclusion sometimes repeats similar ideas, which can be streamlined for clarity and conciseness. For example, the emphasis on the significance of favorable views and the role of social influence is reiterated multiple times. Combining related ideas can improve readability.

Some sentences are complex and could benefit from being broken down into simpler, clearer statements. For instance, the long sentence discussing the importance of promoting favorable views could be divided for better understanding.

Ensure consistent terminology throughout the conclusion. For example, the terms "AI writing tools" and "AI technologies" should be used consistently to avoid confusion.

Proper punctuation helps in clearly separating ideas and ensuring smooth flow. For instance, semicolons can be used to separate closely related independent clauses more effectively.

Watch for minor grammatical issues, such as incorrect verb tenses or awkward phrasing. For example, the phrase "provides actionable insights" could be more precisely phrased to reflect ongoing relevance. Improving these aspects will enhance the overall quality and readability of the conclusion, making the key points more impactful and easier to understand.
