# Supplementary material for: Adoption of AI writing tools among academic researchers: A Theory of Reasoned Action approach
Source: PLoS One. 2025 Jan 9;20(1):e0313837. doi: 10.1371/journal.pone.0313837 (PMC11717249; doi:10.1371/journal.pone.0313837)
Supplement: Attachment — Submitted filename: Respose to reviewer.docx [file pone.0313837.s004.docx]

**Response to Reviewers**

Dear Dr. Dokun Iwalewa OIuwajana and the PLOS ONE Editorial Team,

We sincerely appreciate the time and effort you and the reviewers have dedicated to providing valuable feedback on our manuscript titled ***"Adoption of AI Writing Tools among Academic Researchers: A Theory of Reasoned Action Approach" (PONE-D-24-17396).*** We have carefully considered all the comments and suggestions, and have made substantial revisions to address them. We believe these changes have significantly improved the quality and clarity of our manuscript.

In response to the feedback received, we have made the following key improvements:

1. Overall manuscript quality: We have thoroughly revised the entire manuscript to address grammatical errors, awkward phrasing, and clarity issues. This includes a comprehensive grammar and spell check, revision of phrasing, and review.
2. Introduction and Literature Review: We have clarified the unique contributions of TRA compared to UTAUT, added recent statistics on AI writing tools usage in academia, and provided clearer research questions. We've also expanded our justification for using TRA and included more examples of its application in AI adoption contexts.
3. Methodology: We have provided more detailed rationales for our methodological choices, including sampling strategy and sample size. We've also added information on scale adaptation and data quality procedures.
4. Results and Discussion: We have improved the clarity of our results presentation, including a clearer interpretation of non-significant results. We've also expanded our discussion to include more detailed comparisons with recent AI adoption studies and more specific implications for stakeholders.
5. Conclusion: We have restructured the conclusion to more clearly state our key findings and provide more specific recommendations and future research directions.

For a detailed point-by-point response to each comment, please refer to the attached table. We believe that these revisions have substantially improved our manuscript and addressed all the concerns raised. We have **also highlighted** all changes made to the original version.

Thank you again for your valuable feedback and the opportunity to revise our manuscript. We look forward to your response and are happy to address any further questions or concerns you may have.

Sincerely,

Mohammed A. Al-bukhrani

Corrossponding auther

Table 1 Response to Reviewer’s Comments

| **Section** | **Comment** | **Note** |
| --- | --- | --- |
| Overall | Manuscript contains frequent grammatical errors, awkward phrasing, and some clarity issues that require attention. | Overall grammatical and clarity issues were addressed. |
| Introduction | Clarify the unique contributions of TRA compared to UTAUT in this research. | A detailed paragraph was added comparing TRA and UTAUT. |
|  | Provide more recent statistics on AI writing tools usage in academia. | Recent statistics were added, including a survey about ChatGPT usage. |
|  | Offer a clearer statement of the specific research questions or hypotheses. | Three clear, specific research questions were added at the end of the introduction. |
| Literature Review | Provide a clearer justification for why TRA was chosen over TAM or UTAUT for this specific study. | A more detailed rationale for choosing TRA was provided. |
|  | Offer more examples of how TRA has been specifically applied to AI tools in academic or professional contexts. | Recent studies applying TRA to AI adoption were discussed. |
|  | Consolidate some references to improve readability and flow. | Conolidated where applicable. |
|  | Merge Theory of Reasoned Action and related technologies sections. | The Theory of Reasoned Action and related technologies sections merged. |
| Methodology | Provide a more detailed rationale for why purposive sampling was chosen. | More details on the rationale for purposive sampling were added. |
|  | Discuss how the final sample of 150 compares with similar studies in terms of generalizability. | The sample size was discussed, justified and supported by previose studies applying the same techenque. |
|  | Provide specific examples of how feedback from the pre-test was incorporated. | Examples of how pre-test feedback was incorporated were added. |
|  | Include a brief discussion on the process of adapting scales to the context of AI writing tools. | The process of adapting scales was explained in more detail. |
|  | Provide a clearer explanation of procedures for ensuring data quality and handling incomplete responses. | More details on data quality procedures were added. |
| Results | Clarify that non-significant results indicate a lack of evidence to support hypotheses. | The interpretation of non-significant results was clarified. |
|  | Rewrite the section on H6 in a simpler, more readable way. | The section on H6 was rewritten for clarity. |
|  | Consider additional fit indices or refine the model to achieve a better fit. | The SRMR values were discussed and justified supported by previous studies. |
| Discussion/Implications | Provide a more detailed exploration of why perceived barriers did not significantly impact attitudes or intentions. | A more detailed exploration of this finding was added. |
|  | Compare findings with similar studies in the AI adoption domain. | A detailed comparison with recent AI adoption studies was added. |
|  | Add specific examples of how stakeholders can implement strategies based on the findings. | More specific, actionable recommendations were included for different stakeholders. |
|  | Separate the discussion from the implications. | The paper was restructured to have a separate implications section. |
| Conclusion | Explicitly state the most significant findings at the beginning of the conclusion. | The conclusion now starts with a clear statement of key findings. |
|  | Provide more specific recommendations for stakeholders. | More specific recommendations were added to the conclusion. |
|  | Include a more detailed discussion of potential future research directions. | More detailed future research directions were added. |
|  | Improve grammar, syntax, and overall readability. | The response table doesn't mention specific actions taken to improve the conclusion's grammar and readability. |
